# Clinical characteristics and risk factors of in-hospital mortality among patients undergoing percutaneous pericardiocentesis

**DOI:** 10.3389/fcvm.2023.1252525

**Published:** 2023-09-14

**Authors:** Maciej T. Wybraniec, Zofia Kampka, Mateusz Drabczyk, Marek Zielonka, Przemysław Urbaniec, Grzegorz Wypych, Małgorzata Cichoń, Tomasz Szatan, Paweł Jastrzębski, Katarzyna Mizia-Stec

**Affiliations:** ^1^ First Department of Cardiology, School of Medicine in Katowice, Medical University of Silesia, Katowice, Poland; ^2^ Upper Silesia Medical Centre, Katowice, Poland; ^3^ European Reference Network on Heart Diseases-ERN GUARD-HEART, Amsterdam, Netherlands; ^4^ Department of Cardiology in Cieszyn, Upper-Silesian Medical Centre, Cieszyn, Poland

**Keywords:** pericardiocentesis, cardiac tamponade, in-hospital mortality, registry, pericarditis

## Abstract

**Background:**

Percutaneous pericardiocentesis represents a salvage procedure in case of cardiac tamponade and diagnostic procedure in chronic pericardial effusion of unknown source. The study aimed to analyze the clinical characteristics of patients subject to pericardiocentesis and the predictors of in-hospital mortality.

**Methods:**

The study represents a registry that covered consecutive patients undergoing percutaneous pericardiocentesis from 2011 to 2022 in high-volume tertiary reference center. Electronic health records were queried to obtain demographic and clinical variables. The primary endpoint was in-hospital mortality, while secondary endpoint was the need for recurrent pericardiocentesis.

**Results:**

Out of 132 456 patients hospitalized in the prespecified period, 247 patients were subject to percutaneous pericardiocentesis (53.9% women; median age of 66 years) who underwent 273 procedures. In-hospital death was reported in 14 patients (5.67%), while recurrent pericardiocentesis in 24 patients (9.72%). Iatrogenic cause was the most common etiology (42.5%), followed by neoplastic disease (23.1%) and idiopathic effusion (14.57%). In logistic regression analysis in-hospital mortality was associated with myocardial infarction (MI)-related etiology (*p* = 0.001) and recurrent/persistent cardiogenic shock (*p* = 0.001).

**Conclusions:**

Iatrogenic etiology and neoplastic disease seem to be the most common indications for pericardiocentesis, while in-hospital mortality was particularly high in patients with spontaneous tamponade in the course of MI.

## Introduction

Percutaneous pericardiocentesis belongs to life-saving procedures in cardiac tamponade, while it may also serve as the first line diagnostic procedure in patients with recurrent pericardial effusion (1). The technique usually involves the echocardiography- and fluoroscopy-guided puncture from infrasternal angle, insertion of pig-tail catheter to pericardial cavity, allowing for both *ad hoc* decompression of patients with cardiac tamponade and prolonged drainage of effusion (2). A wide range of clinical indications for the procedure, such as neoplastic disease, chronic kidney disease, viral and autoimmune pericarditis or iatrogenic causes (3–6), make it a universal salvage procedure in cardiology, which should be available in every cardiology department. Given the need for timely intervention in pericardial tamponade, the procedure is often performed by interventional cardiologists in catheterization laboratory. Still paucity of data exists regarding the characteristics and short-term prognosis of patients undergoing this procedure. The aim of the study was to evaluate the clinical characteristics, predictors of in-hospital mortality and in-hospital outcome in patients subject to percutaneous pericardiocentesis.

## Methods

This single-center retrospective study comprised consecutive patients who underwent percutaneous pericardiocentesis between January 2011 and June 2022 regardless of primary diagnosis. The study was performed by means of diligent analysis of electronic health records from the database of Upper–Silesian Medical Center in Katowice, Poland. The patients were selected using procedure class 37.0 according to International Classification of Diseases 9th Edition ICD-9. The study involved both patients subject to urgent pericardiocentesis for tamponade or impeding tamponade or patients undergoing diagnostic pericardial drainage for chronic effusion. Cardiac tamponade was defined a situation, in which symptoms and signs of cardiogenic shock are accompanied by the echocardiographic features of right ventricular and right atrial collapse, ventricular interdependence and more than >50% respiratory fluctuations in maximal velocity of mitral flow. Impeding tamponade represented a clinical scenario of present echocardiographic signs of tamponade without cardiogenic shock. Chronic pericardial effusion was defined as a pericardial effusion of varying extent, which was refractory to medical therapy and was qualified for diagnostic drainage in order to establish specific diagnosis. The analysis included data on demographic parameters, comorbidities, laboratory tests, procedural characteristics and in-hospital mortality. Exclusion criteria involved data incompleteness regarding the etiology and in-hospital outcome, as well as upfront surgical intervention for pericardial fluid. The primary endpoint was in-hospital mortality. The secondary endpoint was recurrent pericardiocentesis. The center technique of percutaneous pericardiocentesis routinely involves the puncture from infrasternal angle under fluoroscopic and echocardiographic guidance, insertion of 6F sheath and introduction of 6F pig-tail catheter over the 0.035 inch J-wire to pericardial cavity. Routine management involves postprocedural treatment with colchicine (0.5 mg twice daily ≥70 kg, 0.5 mg once daily <70 kg for at least 2–4 weeks and non-steroidal anti-inflammatory drugs for at least 2 weeks with further de-escalation of dose in majority of cases given good tolerance of treatment and lack of contraindications. In typical case of viral pericarditis the treatment with colchicine is extended up to 3 months on individual basis (7).

Statistical analysis was performed by means of SPSS v.25.0 software (IBM Corp, Armonk, NY, USA). Distribution of continuous variable was verified using Shapiro–Wilk's test and expressed as median and 1–3 quartile range or mean and standard deviation (SD). All the qualitative parameters were presented as absolute number and percentage. The inter-group differences were compared using Mann–Whitney *U* test (non-normal distribution) or Student's *t*-test (normal distribution) or Chi-square test (qualitative parameters). Univariate analysis of different predictors of in-hospital mortality were established. All the variables with *p* < 0.1 were included in logistic regression analysis.

The study was granted consent by the Ethics Committee of Medical University of Silesia (consent number PCN/CBN/0052/KB/192/22 on 10/08/2022) and the written informed consent was not required due to retrospective and registry-based design.

## Results

**Figure 1:**
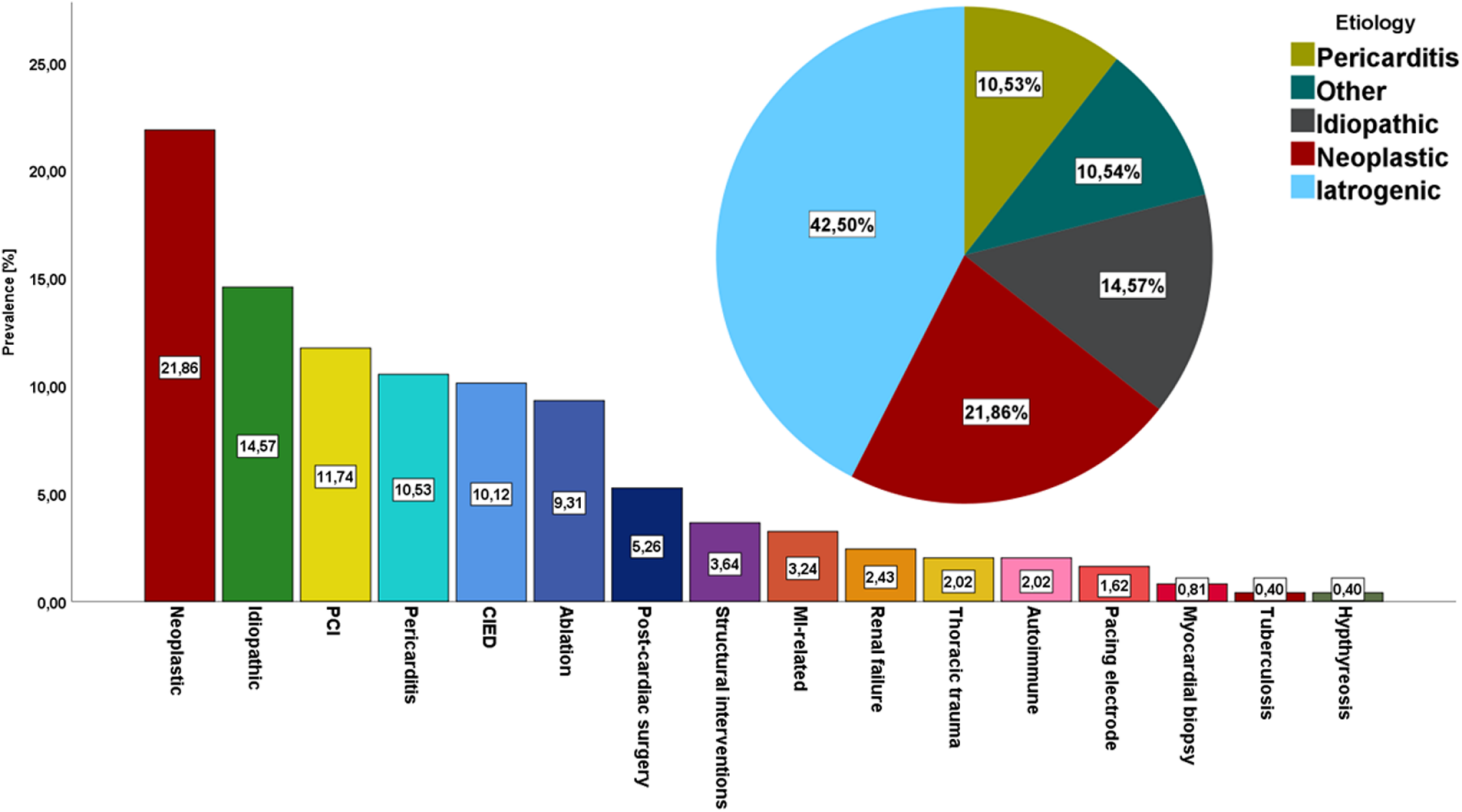
The prevalence of different indications for percutaneous pericardiocentesis:detailed distribution of indications for percutaneous pericardiocentesis and general overview of indications for pericardiocentesis PCI, percutaneous coronary intervention; CIED, cardiac implantable electronic device; MI, myocardial infarction.

Among 132 456 patients hospitalized in the prespecified period, 247 patients were subject to percutaneous pericardiocentesis [133 women, 53.9%; median age of 66 (59; 76) years] who underwent 273 procedures. The amount of patients undergoing pericardiocentesis grew steadily over the study period (Supplementary Figure S1). The baseline demographic and clinical characteristics of study population was presented in Table 1. The median duration of in-hospital stay was 8 (5; 11) days. The majority of percutaneous pericardiocentesis was performed in patients with cardiac tamponade (*n* = 155, 62.75%) or impending tamponade (*n* = 71, 28.74%), while 21 patients underwent diagnostic procedure for chronic pericardial effusion (*n* = 21, 8.5%). The most common indications for percutaneous pericardiocentesis were neoplastic disease (*n* = 54, 23.1%), idiopathic effusion (*n* = 36, 14.57%), coronary artery perforation during percutaneous coronary intervention (*n* = 29, 11.74%), viral pericarditis (*n* = 26, 10.53%), cardiac electronic device implantation (*n* = 25, 10.12%), catheter ablation (*n* = 23, 9.31%) and post-cardiac surgery fluid (*n* = 13, 5.26%), structural interventions (*n* = 9, 3.64%), temporary pacing electrode (*n* = 4, 1.62%), myocardial biopsy (*n* = 2, 0.81%) Out of 9 cases of pericardiocentesis following structural intervention, 7 patients underwent transcatheter aortic valve implantation (77.8%), while 2 underwent left atrial appendage occluder implantation (22.2%). Out of 29 pericardiocenteses following PCI, 25 patients had acute coronary syndrome (86.2%), while 4 chronic coronary syndrome (13.8%). Detailed distribution of indications for pericardiocentesis was highlighted in Figure 1. Overall, iatrogenic cause was the most common indication for PP (*n* = 105, 42.5%). As far as neoplastic disease (*n* = 54) is concerned, the most common source of neoplasm was lung carcinoma (*n* = 30, 55.6%), leukemia or lymphoma (*n* = 6, 11.1%), breast carcinoma (*n* = 4, 7.27%) and other. The source of neoplasm was undetermined in 5 patients (9.26%). Cardiac tamponade was the first sign of neoplastic disease in nearly 1/3 of patients with this etiology (*n* = 18, 34.62%).

**Table 1:** Demographic and clinical characteristics of study population.

Variable	Overall population *N* = 247
Age [years]	66 (59; 76)
Weight [kg]	75 (64; 92)
Female sex	133 (53.9%)
Duration of initial hospitalization [days]	8 (5; 11)
Arterial hypertension	145 (59.7%)
Diabetes mellitus/IFG/IGT	56 (22.7%)
Chronic obstructive pulmonary disease	13 (5.3%)
History of MI	37 (15.0%)
History of PE	6 (2.4%)
History of TIA/stroke	17 (6.9%)
Hypothyroidism	32 (13.0%)
Cigarette smoking	57 (23.1%)
Active COVID-19	9 (3.6%)
Oral anticoagulation	50 (20.2%)
SBP initial [mmHg]	125.3 ± 27.0
DBP initial [mmHg]	74.4 ± 15.5
SBP min. [mmHg]	97.3 ± 24.3
DBP min. [mmHg]	58.4 ± 13.9
LVEF [%]	55 (50; 60)
Left atrial diameter [mm]	37 (33; 42)
CRP [mg/L]	31.7 (9.1; 90.8)
Hgb—baseline [g/dl]	12.3 ± 1.8
Hgb—min [g/dl]	11.3 ± 2.5
WBC [×1000/mm^3^]	9.2 (7.1; 12.3)
PLT [×1000/mm^3^]	237 (180; 308)
ALAT [U/L]	28 (17; 55)
K + [mmol/L]	4.3 ± 0.6
Na + [mmol/L	137.9 ± 5.2
Fasting glucose [mg/dl]	107 (92; 133)
SCr [mg/dl]	1.0 (0.8; 1.3)
TSH [uIU/ml]	1.5 (0.9; 3.1)
LDH [U/L]	212 (174; 537)
Urgent pericardiocentesis	226 (91.5%)
Pericardial fluid drainage [ml]- 1	550 (310; 850)
Pericardial fluid drainage [ml]- 2	482.5 (390; 800)
Pericardial fluid drainage [ml]- 3	560 (280; 840)
Pig-tail catheter at discharge	31 (12.6%)
Periprocedural complications	13 (5.3%)
Unsuccessful pericardiocentesis	8 (3.2%)
Surgical intervention following pericardiocentesis	18 (7.3%)
Persistent cardiogenic shock	33 (13.4%)
New-onset atrial fibrillation	77 (31.6%)
Transfer to ICU	29 (11.8%)
Recurrent pericardiocentesis	24 (9.7%)
Triple pericardiocentesis	2 (0.8%)
Need for packed red blood cells transfusion	28 (11.3%)
Need for fresh frozen plasma transfusion	6 (2.4%)
In-hospital death	14 (5.7%)

ALAT, alanine transaminase; MI, myocardial infarction; PE, pulmonary embolism; TIA, transient ischemic attack; Hgb, hemoglobin; LVEF, left ventricular ejection fraction; CRP, C-reactive protein; SBP, systolic blood pressure; DBP, diastolic blood pressure; IFG, impaired fasting glucose; IGT, impaired glucose tolerance; ICU, intensive care unit; LDH, lactic dehydrogenase; WBC, white blood cell count; PLT, platelet count; SCr, serum creatinine concentration; TSH, thyroid-stimulating hormone.

The median volume of pericardial fluid was 550 (310; 850) ml. The analysis of drainage showed hemorrhagic effusion in 126 patients (51.0%), which was equally distributed between neoplastic and non-neoplastic etiologies (*p* = 0.482), while atypical cells were reported in 40% patients with neoplastic disease and 10.8% of patients with non-neoplastic indication for pericardiocentesis (*p* = 0.001). Of note, 60% of patients with malignant neoplasms did not exhibit atypical cells in pericardial effusion.

The comparison between iatrogenic and non-iatrogenic indications for pericardiocentesis in terms of demographic and clinical variables is highlighted in Table 2.

**Table 2:** Demographic and clinical variables depending on etiology: iatrogenic vs. non-iatrogenic indication for pericardiocentesis.

Variable	Non-iatrogenic *N* = 142	Iatrogenic *N* = 105	*P*-value
Age [years]	64.5 (55; 74)	70 (60; 80)	0.003
Weight [kg]	74 (60; 91.5)	75 (68; 91)	0.441
Female sex	73 (51.4%)	60 (57.1%)	0.371
Duration of initial hospitalization [days]	7 (4; 10)	8 (6; 13)	0.003
Arterial hypertension	70 (49.3%)	75 (71.4%)	0.001
Diabetes mellitus/IFG/IGT	32 (22.5%)	24 (22.9%)	0.952
Chronic obstructive pulmonary disease	10 (7.0%)	3 (2.9%)	0.132
History of MI	15 (10.6%)	22 (21.0%)	0.028
History of PE	4 (2.8%)	2 (1.9%)	0.627
History of TIA/stroke	7 (4.9%)	10 (9.5%)	0.173
Hypothyroidism	13 (9.2%)	19 (18.1%)	0.045
Cigarette smoking	40 (29.9%)	17 (16.5%)	0.017
Active COVID-19	6 (4.3%)	3 (2.9%)	0.549
Oral anticoagulation	16 (11.3%)	34 (32.4%)	<0.001
SBP initial [mmHg]	125.0 ± 25.8	125.6 ± 28.6	0.960
DBP initial [mmHg]	74.6 ± 16.9	74.2 ± 14.0	0.558
SBP min. [mmHg]	103.5 ± 20.3	90.7 ± 26.6	0.019
DBP min. [mmHg]	60.6 ± 13.6	56.0 ± 14.0	0.052
LVEF [%]	55 (50; 60)	55 (45; 60)	0.311
Left atrial diameter [mm]	36 (32; 40)	38 (35; 43)	0.005
CRP [mg/L]	30.3 (8.8; 82.2)	34.0 (11.2; 133.3)	0.172
Hgb—baseline [g/dl]	12.2 ± 1,8	12.6 ± 1.9	0.055
Hgb—min [g/dl]	11.6 ± 2.9	10.9 ± 1.8	0.039
WBC [×1000/mm^3^]	9.83 (7.51; 13.1)	8.49 (6.67; 11.38)	0.012
PLT [×1000/mm^3^]	258 (184; 326)	209.5 (176.5; 262.5)	0.001
ALAT [U/L]	31 (17; 61)	24.5 (16.5; 46.5)	0.227
K + [mmol/L]	4.27 ± 0.66	4.28 ± 0.55	0.988
Na + [mmol/L	136.9 ± 5.8	139.3 ± 3.8	0.002
Fasting glucose [mg/dl]	114 (92; 141)	104 (92; 120)	0.055
SCr [mg/dl]	0.98 (0.76; 1.41)	0.99 (0.83; 1.31)	0.280
TSH [uIU/ml]	1.66 (0.91; 3.2)	1.51 (0.94; 2.72)	0.706
LDH [U/L]	202 (162; 537)	351 (205.5; 571)	0.339
Urgent pericardiocentesis	122 (85.9%)	105 (100%)	<0.001
Pericardial fluid drainage [ml]- 1	675 (400; 900)	350 (250; 630)	<0.001
Periprocedural complications	7 (4.9%)	7 (6.7%)	0.569
Unsuccessful pericardiocentesis	6 (4.2%)	2 (1.9%)	0.308
Persistent cardiogenic shock	12 (8.5%)	21 (20.0%)	0.008
New-onset atrial fibrillation	33 (23.2%)	44 (41.9%)	0.002
Transfer to ICU	15 (10.6%)	14 (13.3%)	0.486
Recurrent pericardiocentesis	18 (12.7%)	6 (5.7%)	0.068
Need for packed red blood cells transfusion	14 (9.9%)	14 (13.3%)	0.395
Need for fresh frozen plasma transfusion	3 (2.1%)	3 (2.9%)	0.707
In-hospital death	10 (7.0%)	4 (3.8%)	0.277

ALAT, alanine transaminase; MI, myocardial infarction; PE, pulmonary embolism; TIA, transient ischemic attack; Hgb, hemoglobin; LVEF, left ventricular ejection fraction; CRP, C-reactive protein; SBP, systolic blood pressure; DBP, diastolic blood pressure; IFG, impaired fasting glucose; IGT, impaired glucose tolerance; ICU, intensive care unit; LDH, lactic dehydrogenase; WBC, white blood cell count; PLT, platelet count; SCr, serum creatinine concentration; TSH, thyroid-stimulating hormone; min., minimal.

Periprocedural complications other than death, were reported in 13 patients (5.26%), including pneumothorax (*n* = 2, 0.8%), pleural bleeding (*n* = 1, *n* = 0.4%), myocardial wall perforation (*n* = 3, 1.21%), local infection (*n* = 1, 0.4%), sudden cardiac arrest (*n* = 6, 2.43%). Unsuccessful percutaneous pericardiocentesis was documented in 8 patients (3.24%), while conversion to surgical intervention was necessary in 17 patients (7.29%). Persistent cardiogenic shock following the initial pericardiocentesis was documented in 33 patients (13.36%), whereas 29 patients were transferred to intensive care unit (11.79%). Atrial fibrillation was confirmed in 77 patients (31.56%).

In-hospital death was reported in 14 patients (5.67%). The mortality rate differed across different indications for pericardiocentesis. In-hospital mortality was the highest in patients undergoing pericardiocentesis due to myocardial infarction-related myocardial rupture (*n* = 5, 62.5%), in chest trauma (*n* = 1, 20%), autoimmune pericarditis (*n* = 1, 20%), followed by post-cardiac surgery tamponade (*n* = 1, 7.7%), percutaneous coronary intervention-related coronary artery perforation (*n* = 2, 6.9%), cardiac implantable electronic device-related tamponade (*n* = 1, 4%), viral pericarditis (*n* = 1, 3.8%), idiopathic pericardial fluid (*n* = 1, 2.8%), and neoplastic disease (*n* = 1, 1.9%; *p* < 0.0001). Patients with iatrogenic indication for pericardiocentesis had similar in-hospital mortality to other etiologies (3.81% vs. 7.04%, *p* = 0.277). No cases of death were reported in patients with pericardiocentesis related with cardiac ablation and electrophysiology procedures (overall cohort of *n* = 23). Univariate analysis revealed that in-hospital death was predicted by myocardial infarction-related etiology, persistent cardiogenic shock in the course of hospitalization, lower pericardial drainage volume, right bundle branch block, packed red blood cells transfusion (Table 3). Logistic regression indicated that myocardial infarction-related etiology (OR 44.3, 95% CI: 4.58–428.94, *p* = 0.001) and recurrent/persistent cardiogenic shock (OR 12.3, 95% CI: 2.71–55.95, *p* = 0.001) were independently associated with in-hospital mortality [area under curve for the model (AUC) 0.793; 95% CI: 0.723–0.853, Hosmer–Lemeshow *p* = 1.0].

**Table 3:** Univariate and logistic regression analysis of different predictors of in-hospital death in patients undergoing percutaneous pericardiocentesis.

Variable	OR	95% CI	*P*
Volume of drainage [per 1 ml]	0.997	0.995–0.999	0.0141
Age [per 1 year]	1.05	1.004–1.105	0.0347
Duration of pericardial drainage [per 1 day]	0.59	0.37–0.955	0.0316
LVEF [per 1%]	0.96	0.92–0.996	0.0303
Pericarditis-related pericardiocentesis	8.89	0.76–104.48	0.0824
MI-related cardiac tamponade	42.5	8.78–206.52	<0.0001
Unsuccessful pericardiocentesis	6.31	1.15–34.60	0.0340
Complications of pericardiocentesis	6.06	1.46–25.18	0.0133
RBBB	5.43	1.32–22.31	0.0189
Persistent cardiogenic shock after pericardiocentesis	5.72	1.84–17.75	0.0025
Transfer to ICU	3.31	0.97–11.35	0.0567
Need for packed red blood cells transfusion	3.48	1.01–11.97	0.0475
Logistic regression			
MI-related cardiac tamponade	19.0	2.36–152.84	0.006
Persistent cardiogenic shock after pericardiocentesis	12.3	2.71–55.95	0.001
Area under ROC 0.793; 95% CI: 0.723–0.853; Hosmer–Lemeshow *p* = 1.0			

RBBB, right bundle branch block; LVEF, left ventricular ejection fraction; MI, myocardial infarction; ICU, intensive care unit; OR, odds ratio; CI, confidence interval; ROC, receiver operating characteristics curve.

In the subgroup of patients with iatrogenic indication for pericardiocentesis, univariate analysis indicated that requirement for fresh frozen plasma transfusion (OR 16.5, 95% CI: 1.15–236.16; *p* = 0.039), complications of pericardiocentesis (OR 19.2, 95% CI: 2.22–165.88; *p* = 0.007) were significantly associated with in-hospital death, while age (OR 1.1 per 1 year; 95% CI: 0.98–1.23. *p* = 0.092) and persistent cardiogenic shock (OR 4.32, 95% CI: 0.57–32.62, *p* = 0.157) had borderline significance towards prediction of in-hospital death. Logistic regression analysis confirmed that the presence of complications of pericardiocentesis (OR 19.2, 95% CI: 2.22–165.88, *p* = 0.007) was the only independent predictor of in-hospital death among patients with iatrogenic cause of tamponade (AUC 0.725; 95% CI 0.63–0.81, Hosmer–Lemeshow *p* = 1.0).

In the cohort of patients with non-iatrogenic requirement for pericardiocentesis, univariate analysis showed that persistent cardiogenic shock (OR 10.3, 95% CI: 2.42–44.19, *p* = 0.002), myocardial infarction-related myocardial rupture (OR 43.0, 95% CI: 7.96–232.41, *p* < 0.0001), left ventricular ejection fraction (OR 0.92 per 1% increase, 95% CI: 0.86–0.97, *p* = 0.005), volume of pericardial drainage (OR 0.99, 95% CI: 0.990–0.999, *p* = 0.01) predicted in-hospital mortality. There was a trend towards association between in-hospital mortality and the need for packed red blood cells transfusion (OR 4.71, 95% CI: 1.07–20.85, *p* = 0.041) and history of sudden cardiac arrest (OR 5.13, 95% CI: 0.89–29.58, *p* = 0.068) and right bundle branch block (OR 7.81, 95% CI: 1.24–49.28, *p* = 0.029) and neoplastic etiology (OR 0.17, 95% CI: 0.02–1.35, *p* = 0.093). In the logistic regression analysis, persistent cardiogenic shock after pericardiocentesis (OR 66.4, 95% CI: 6.21–710.26, *p* = 0.001) and myocardial infarction-related tamponade (OR 83, 95% CI: 5.15–1338.14, *p* = 0.002) represented independent predictors of in-hospital mortality (AUC 0.891, 95% CI: 0.812–0.945, Hosmer–Lemeshow *p* = 1.0).

Recurrent pericardiocentesis was performed in 24 patients (9.72%), of which 11 were performed during the same hospitalization (45.83%), while 13 required readmission to hospital (54.17%). Triple pericardiocentesis was reported in 2 patients (0.81%). The population of patients with recurrent need for pericardiocentesis was characterized by a trend towards lower rate of iatrogenic etiology (25% vs. 44.4%, *p* = 0.068) and a tendency to higher rate of neoplastic etiology (33.3% vs. 20.6%, *p* = 0.152), greater prevalence of men (66.67% vs. 43.95%, *p* = 0.034), greater median volume of drained fluid during initial procedure (800 vs. 535 ml, *p* = 0.034) and a trend towards younger age (63.5 vs. 67 years, *p* = 0.070) and greater concentration of hemoglobin (13.23 vs. 12.25 g/dl, *p* = 0.018). Logistic regression analysis showed that iatrogenic etiology was a negative predictor (OR 0.36, 95% CI: 0.13–0.98, *p* = 0.462), while volume of drained fluid was a positive predictor (unit OR per 10 ml 1.34; 95% CI 1.12–1.52, *p* = 0.036) of the need for recurrent pericardiocentesis (AUC 0.674; 95% CI: 0.610–0.734, Hosmer–Lemeshow *p* = 0.423).

## Discussion

The results of the present research should be confronted with the biggest study by Gad MM et al. performed on the cohort of 96 377 patients subject to pericardiocentesis between 2007 and 2015 (8). Gad MM et al. found that the mortality was nearly twice as high as in the current research, reaching 13.1% (8). Also, in contrast to our report, the risk of death was linked to structural heart disease-related tamponade, bacterial pericarditis and neoplastic disease (8). In our study the myocardial infarction-related tamponade was characterized by the highest risk of mortality, while no cases of death were reported in 9 patients who experienced cardiac tamponade in the course of structural interventions. Also, in our study neoplastic disease was not independently associated with increased risk of in-hospital death, but a trend towards higher rate of recurrent pericardiocentesis was reported in this subset of patients. Although pericardial involvement represents marker of a late stage of malignant neoplasm, it often translates into high mortality following hospital discharge and poor long-term outcome.

Sethi and coworkers evaluated the in-hospital mortality in 64,070 patients treated with pericardiocentesis on the basis of data from Nationwide Inpatient Sample and also documented much higher in-hospital mortality of 12.3% than in present report (8). The Authors found that the mortality was particularly high in patients submitted to pericardiocentesis after percutaneous coronary intervention, structural interventions and cardiac surgery (9). One should note that this research showed considerably lower prevalence of iatrogenic indications for pericardiocentesis (17.7%), which might be conditioned by lower amount of procedures performed in years 2007-2013 in comparison to present times (9).

The finding of particularly high prevalence of neoplastic pericardial effusion as an indication for pericardiocentesis is consistent with other reports, including the study by Pawlak–Cieslik et al., who documented that malignant pericarditis was present in 58% of patients subject to pericardial drainage (5). In the cited study lung carcinoma represented the most common type of neoplasm responsible for pericardial effusion (67%) (5), similarly to our report (55.6%).

A discrepancy between indications for pericardiocentesis may be related with the profile of the analyzed center. In the study by Del Portillo–Navarrete JH et al, 26.7% of all patients underwent pericardiocentesis on account of post-pericardiotomy syndrome (10), whereas in our report this indication was responsible for roughly 5% of patients.

A single center report by Pennacchioni A et al. also demonstrated mortality of 14.8%, which was heralded by non-neoplastic/non-idiopathic etiology and cardiogenic shock (11). All in all, the overview of the literature showed disparity in terms of risk factors of mortality, which might be conditioned by different methodology (high-volume institutional registries vs. single center reports) and time of data collection. Our study cohort was characterized by nearly twice lower in-hospital mortality than previously reported. Also, unlike previous reports (8–11) iatrogenic indications for pericardiocentesis were not associated with increased risk of death. Our study recapitulated former observations that refractory hemodynamic instability following pericardial decompression is linked to impaired in-hospital outcome (11). For the first time our study provided data on the rate and predictors of the need for recurrent pericardiocentesis, which is particularly frequent in patients with large volume of initial pericardial drainage and non-iatrogenic indication for the procedure. The need for recurrent pericardiocentesis was much lower in our study than in the similar study by Cheong XP and coworkers, who found especially high risk of recurrence in patients with malignant neoplasms as a cause of tamponade (12).

In our study complications of the pericardiocentesis occurred in 13 patients (5.26%), which is slightly higher than data in literature (8). Also, given the hemorrhagic nature of iatrogenic tamponade, 28 patients (11.3%) required packed red blood cell transfusion. It is worth to mention that this complication can be easily managed by autologous blood transfusion, which represents a promising technique of blood sparing, yet it has not been implemented at our institution (13).

Potential study limitations involve retrospective and single-center study design, which introduce significant selection bias and limit wide application of the results of the study. The profile of a center (proportion of cardiac surgical vs. non-surgical indications) affects the proportion of patients with different etiologies of pericardial effusion. Some cases of *ad hoc* pericardiocentesis performed outside of catheterization laboratory might have been underreported. In our center primarily infrasternal approach was used, while no primary surgical interventions were included. The study did not provide data on post-discharge prognosis of patients, other than the need for recurrent pericardiocentesis.

## Conclusions

Based on current data, iatrogenic etiology represents the most frequent indication for percutaneous pericardiocentesis, followed by neoplastic disease. Pericardial effusion requiring pericardiocentesis may be the first sign of neoplastic disease and oncological caution is warranted. The diagnosis of non-iatrogenic tamponade or impeding tamponade, especially with hemorrhagic effusion should prompt further use of imaging modalities in order to exclude neoplastic disease. Myocardial rupture in the course of myocardial infarction and persistent cardiogenic shock confer the greatest risk of in-hospital death. Complications of electrophysiology procedures constitute a frequent indication for pericardiocentesis, yet they are related with low risk of in-hospital death given proper management. Clinicians should inform patients qualified for elective electrophysiology procedures that the prognosis is good even if cardiac tamponade occurs. Nearly 1 out of 10 patients requires recurrent pericardiocentesis, which is particularly frequent in patients with greater baseline volume of pericardial fluid and non-iatrogenic etiology. In this case longer maintenance of pig-tail catheter in the pericardial cavity should be considered with the intention to avoid the need for recurrent puncture.

## Data Availability

The raw data supporting the conclusions of this article will be made available by the authors, without undue reservation.

## Supplementary material

Supplementary Figure S1 The annual number or pericardiocentesis in the time between 2011 and 2022.
