# Supplementary figures and images for: Clinical characteristics and risk factors of in-hospital mortality among patients undergoing percutaneous pericardiocentesis
Source: Front Cardiovasc Med. 2023 Sep 14;10:1252525. doi: 10.3389/fcvm.2023.1252525 (PMC10537933; doi:10.3389/fcvm.2023.1252525)

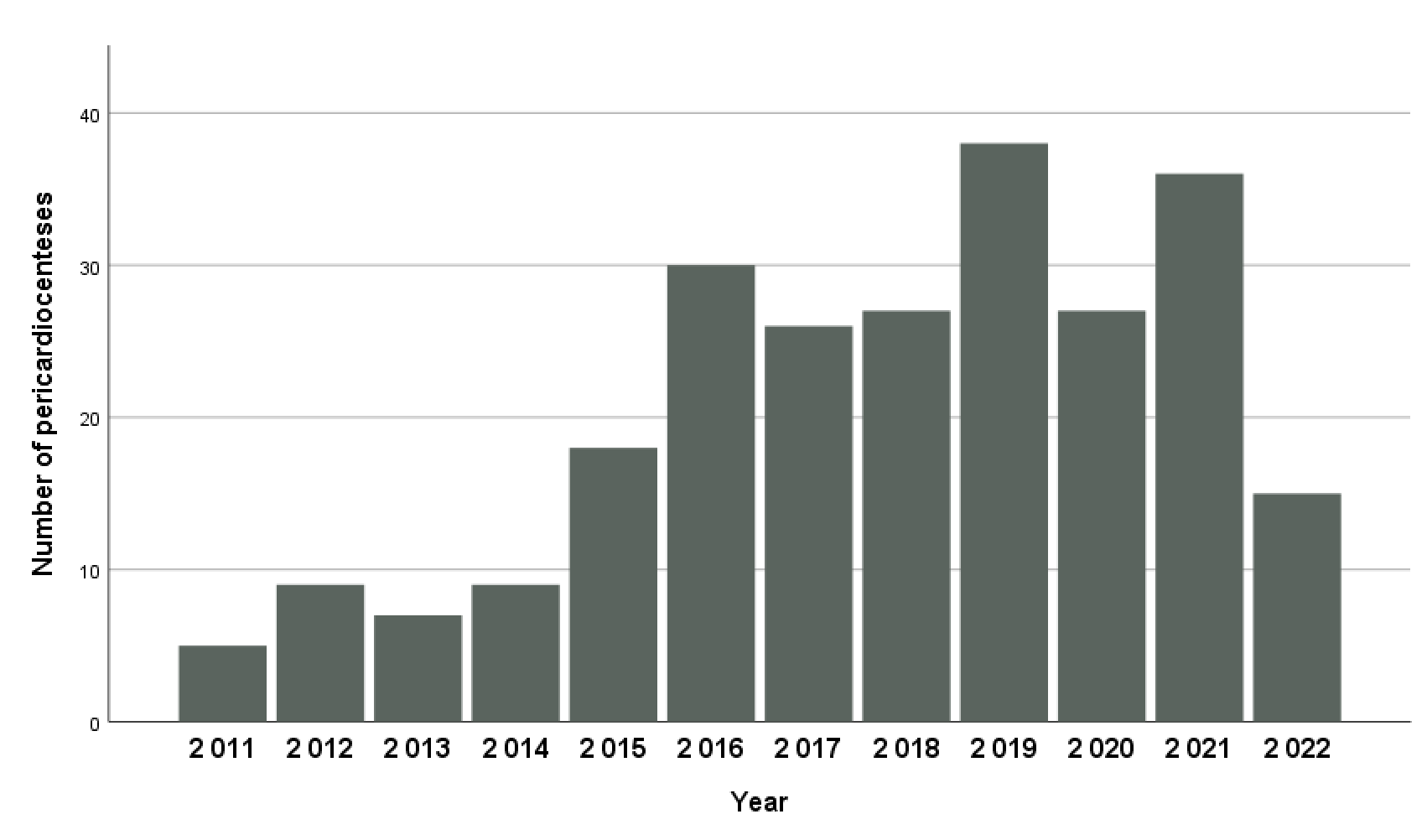

Supplement: Supplementary Figure S1 — The annual number or pericardiocentesis in the time between 2011 and 2022. [file Image1.tif]
